# Supplementary material for: In Vitro Proof-of-Concept Study: Lidocaine and Epinephrine Co-Loaded in a Mucoadhesive Liquid Crystal Precursor System for Topical Oral Anesthesia
Source: Pharmaceuticals (Basel). 2025 Aug 6;18(8):1166. doi: 10.3390/ph18081166 (PMC12389179; doi:10.3390/ph18081166)
Supplement: Supplementary file 1 [file pharmaceuticals-18-01166-s001.zip › pharmaceuticals-3698712-supplementary.pdf]

In Vitro Proof-of-Concept Study: Lidocaine and Epinephrine Co-loaded in a Mucoadhesive Liquid Crystal Precursor System for Topical Oral Anesthesia

Giovana Maria Fioramonti Calixto[1], Aylla Mesquita Pestana[1], Arthur Antunes Costa Bezerra[1], Marcela Tavares Luiz[2], Jonatas Lobato Duarte[2], Marlus Chorilli[2], Michelle Franz-Montan[1*]

[1] Department of Biosciences. Piracicaba Dental School. University of Campinas – UNICAMP. Piracicaba. São Paulo. Brazil.

[2] Department of Drugs and Medicines. School of Pharmaceutical Sciences. São Paulo State University - UNESP. 14800-903. Araraquara. Sao Paulo. Brazil.

* Corresponding author:

Michelle Franz-Montan

Piracicaba Dental School. Universidade Estadual de Campinas. UNICAMP

Av. Limeira. 901 Bairro Areião. Piracicaba. SP. Brazil

E-mail: mfranz@unicamp.br

Supplementary material

Table S1- Composition (%) of the liquid crystalline precursor systems (LCPS), active compounds (lidocaine and epinephrine) and saliva-diluted formulations.

Formulation	PPG-5-CETETH-20 (%, w/w)	Oleic Acid (%, w/w)	0.5% (w/v) Chitosan Dispersion (%, w/w)	Lidocaine (%, w/w)	Epinephrine (%, w/w)	Artificial Saliva Addition (%, w/w)
F	40	30	30	–	–	-
F30	40	30	30	–	–	30
F100	40	30	30	–	–	100
FL	40	30	30	5	–	-
FL30	40	30	30	5	–	30
FL100	40	30	30	5	–	100
FLE	40	30	30	5	0.001	-
FLE30	40	30	30	5	0.001	30
FLE100	40	30	30	5	0.001	100
FC	–	–	–	5	–	-
FC30	–	–	–	5	–	30
FC100	–	–	–	5	–	100

Table S2 - Values of q_{max}(Å) and ratio between interplanar distances for formulations. **F** is the liquid crystal precursor system without the incorporation of lidocaine and epinephrine. **FL** is the liquid crystal precursor system with 5% lidocaine hydrochloride. **FLE** is the liquid crystal precursor system with 5% lidocaine hydrochloride and 0.001% epinephrine. The formulations were diluted with 30% (F30, FL30, FLE30) and 100% (F100, FL100, FLE100) of artificial saliva.

Formulations	q_{max1}	q_{max2}	q_{max3}	d_1/d_2	d_1/d_3	Structure	Classification by PLM
**F**	0.08	-	-	-	-	Microemulsion	Microemulsion
**F30**	0.07	0.14	0.21	2	3	Lamellar	Hexagonal
**F100**	0.06	0.12	-	2	-	Hexagonal	Cubic
**FL**	0.08	-	-	-	-	Microemulsion	Microemulsion

**FL30**	0.06	0.12	0.17	2	2.8	Hexagonal	Lamellar
**FL100**	0.06	0.12	-	2	-	Lamellar	Cubic
**FLE**	0.08	-	-	-	-	Microemulsion	Microemulsion
**FLE30**	0.07	0.14	0.21	2	3	Lamellar	Lamellar
**FLE100**	0.05	0.10	1.95	2	3	Lamellar	Cubic

Table S3 - The effects of formulation and artificial saliva on apparent viscosity, flow index, yield strength, and thixotropy. **F** is the liquid crystal precursor system without the incorporation of lidocaine and epinephrine. **FL** is the liquid crystal precursor system with 5% lidocaine hydrochloride. **FLE** is the liquid crystal precursor system with 5% lidocaine hydrochloride and 0.001% epinephrine. **FC** is the commercial formulation of lidocaine 50 mg/g EMS orange flavor dermatological ointment. The formulations were diluted with 30% (F30, FL30, FLE30, FC30) and 100% (F100, FL100, FLE100, FC100) of artificial saliva.

Formulations	Consistency Index ($Pa.s^n$)	Flow index (admensional)	Yield strength (Pa)	Thixotropy (Pa/s)
**F**	1.53 ± 0.15 ^{aA}	0.69 ± 0.06 ^{aA}	1.25 ± 0.13 ^{aA}	363.76 ± 52.30 ^{aA}
**F30**	8.37 ± 1.11 ^{aA}	0.47 ± 0.02 ^{bA}	9.01 ± 1.22 ^{bA}	796.68 ± 34.07 ^{aA}
**F100**	133.89 ± 10.49 ^{aA}	0.14 ± 0.04 ^{cA}	29.77 ± 2.15 ^{cA}	2,425.72 ± 373.30 ^{bA}
**FL**	0.67 ± 0.01 ^{aA}	0.81 ± 0.00 ^{aB}	0.77 ± 0.04 ^{aA}	89.29 ± 4.38 ^{aA}
**FL30**	5.99 ± 0.67 ^{aA}	0.38 ± 0.08 ^{bB}	4.27 ± 0.60 ^{aB}	253.33 ± 48.46 ^{aB}
**FL100**	239,371.56 ± 7,047.64 ^{bB}	0.00 ± 0.00 ^{cB}	63.20 ± 6.04 ^{bB}	10,780.95 ± 307.05 ^{bB}
**FLE**	0.49 ± 0.03 ^{aA}	0.79 ± 0.01^{aB}	0.31 ± 0.05 ^{aA}	100.19 ± 7.16 ^{aA}
**FLE30**	15.32 ± 1.24 ^{aA}	0.16 ± 0.14 ^{bC}	6.12 ± 0.78 ^{bAB}	446.78 ± 57.84 ^{aAB}
**FLE100**	129,132.44 ± 7,047.64 ^{bC}	0.00 ± 0.00 ^{cB}	33.34 ± 4.26 ^{cA}	7,794.04 ± 932.40 ^{bC}
**FC**	34,772.25 ± 1,015.15 ^{aB}	0.00 ± 0.00 ^{aC}	21.71 ± 2.92 ^{aB}	1,543.23 ± 141.35 ^{aB}
**FC30**	0.04 ± 0.00 ^{bA}	0.99 ± 0.01 ^{bD}	0.07 ± 0.01 ^{bC}	11.23 ± 0.44 ^{bC}
**FC100**	0.04 ± 0.00 ^{bA}	0.99 ± 0.01 ^{bC}	0.04 ± 0.00 ^{bC}	10.55 ± 0.90 ^{bD}

Different lowercase letters show a statistical difference between the percentages of artificial saliva incorporated within the same formulation group ($p<0.05$ Bonferroni). Different capital letters show a statistical difference between formulations within the same group of percentage of artificial saliva ($p<0.05$ Bonferroni).

Table S4 - Elastic modulus (G'). loss tangent (tan δ.) viscoelastic exponent (n) and gel strength of the formulations. **F** is the liquid crystal precursor system without the incorporation of lidocaine and epinephrine. **FL** is the liquid crystal precursor system with 5% lidocaine hydrochloride. **FLE** is the liquid crystal precursor system with 5% lidocaine hydrochloride and 0.001% epinephrine. **FC** is the commercial formulation of lidocaine 50 mg/g EMS orange flavor dermatological ointment. The formulations were diluted with 30% (F30, FL30, FLE30, FC30) and 100% (F100, FL100, FLE100, FC100) of artificial saliva.

Formulations	G' (Pa)	Loss tangent (tan δ)	n	Gel strenght (Pa.s)
**F**	18.68 ± 6.18 ^{aA}	0.62 ± 0.11 ^{aA}	0.33 ± 0.10 ^{aA}	16.86 ± 6.38 ^{aA}
**F30**	208.87 ± 12.91 ^{bAB}	0.24 ± 0.00 ^{aA}	0.22 ± 0.01 ^{bA}	205.78 ± 12.89 ^{bA}
**F100**	414.38 ± 67.36 ^{cA}	0.27 ± 0.03 ^{aA}	0.12 ± 0.04 ^{cA}	395.02 ± 70.95 ^{cA}
**FL**	4.44 ± 0.31 ^{aA}	1.19 ± 0.07 ^{aA}	0.44± 0.01 ^{aB}	3.644 ± 0.22 ^{aA}
**FL30**	126.10 ± 14.23 ^{bB}	0.30 ± 0.03 ^{aA}	0.16 ± 0.03 ^{bA}	123.05 ± 15.01 ^{bB}
**FL100**	1038.55 ± 94.99 ^{cB}	0.11 ± 0.02 ^{aA}	0.02 ± 0.00 ^{cB}	1.037.714 ± 94.62 ^{cB}
**FLE**	1.58 ± 0.11 ^{aA}	3.64 ± 0.19 ^{aB}	1.01 ± 0.07 ^{aC}	0.623 ± 0.10 ^{aA}
**FLE30**	181.88 ± 19.46 ^{bB}	0.28 ± 0.01 ^{bA}	0.13 ± 0.01 ^{bA}	178.78 ± 19.40 ^{bAB}
**FLE100**	612.60 ± 31.55 ^{cC}	0.09 ± 0.01 ^{bA}	0.02 ± 0.00 ^{cB}	612.14 ± 31.41 ^{cC}
**FC**	294.04 ± 35.91 ^{aB}	0.58 ± 0.01 ^{aA}	0.25 ± 0.01 ^{aA}	275.95 ± 34.30 ^{aB}
**FC30**	0.43 ± 0.03 ^{bC}	3.00 ± 1.35 ^{bB}	1.72 ± 0.12 ^{bB}	0.034 ± 0.005 ^{bC}
**FC100**	0.41 ± 0.04 ^{bD}	25.60 ± 3.82 ^{cB}	1.91 ± 0.07 ^{cC}	0.033 ± 0.003 ^{bD}

Different lowercase letters show a statistical difference between the percentages of artificial saliva incorporated within the same formulation group ($p<0.05$ Bonferroni). Different capital letters show a statistical difference between formulations within the same percentage of artificial saliva ($p<0.05$ Bonferroni).

Table S5 - Comparative summary of physicochemical, rheological, and functional properties of the developed liquid crystal precursor system (FLE) and the commercial lidocaine formulation (FC).

Parameter	Developed Formulation (FLE)	Commercial Formulation (FC)
Viscosity behavior	Higher viscosity; pseudoplastic behavior	Lower viscosity
Rheological profile	Non-Newtonian, shear-thinning	Newtonian or weakly shear-thinning
Mechanical properties	Higher hardness and adhesiveness	Lower mechanical resistance
Mucoadhesion	Greater mucoadhesive force and work	Lower mucoadhesive performance
Drug release	Sustained release profile; slower than FC	Faster release profile
Permeation	Higher permeation flux and drug retention	Lower permeation and retention
Toxicity classification	Moderate irritant	Moderate irritant

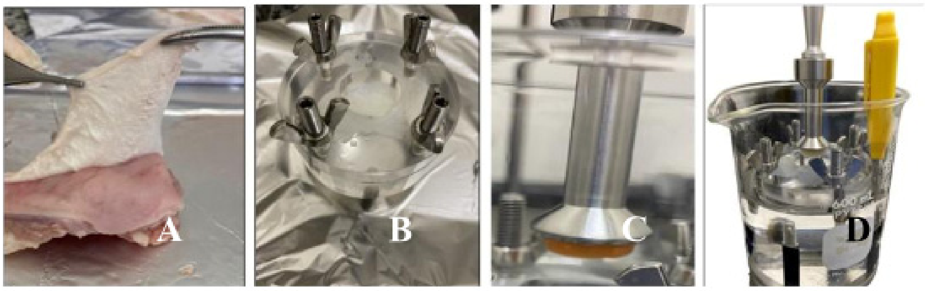

Figure S1 – Preparation of porcine buccal mucosa for the mucoadhesion test: (A) separation of the epithelium from adjacent tissues; (B) epithelium sample fixed to the mucoadhesion device (A/MUC); (C) formulation fixed to the A/MUC probe; (D) formulation and support immersed in artificial saliva (pH 6.8) at 37 °C.
